# Pluripotent stem cell–derived extracellular vesicles for systemic immune modulation in diabetes therapy

**DOI:** 10.21203/rs.3.rs-6415252/v1

**Published:** 2025-06-10

**Authors:** Song Li, Jana Zarubova, Mohammad Hasani-Sadrabadi, Yutong Wu, Graciel Diamante, Jenny Cheng, Xiao Han, Fatemeh Majedi, Li Yang, Olivia Wang, In sook Ahn, Jianyi Zhang, Xiaojun Lian, Zhen Gu, Manish Butte, Reza Ardehali, Peter Butler, Tony Hu, Louis Bouchard, Xia Yang

**Affiliations:** UCLA; University of California, Los Angeles; University of California, Los Angeles; University of California, Los Angeles; University of California, Los Angeles; University of California, Los Angeles; University of California, Los Angeles; University of California, Los Angeles; Center for Cellular and Molecular Diagnostics, Department of Biochemistry and Molecular Biology, Tulane University School of Medicine; University of California, Los Angeles; University of California, Los Angeles; University of Alabama at Birmingham; Pennsylvania State University; State Key Laboratory of Advanced Drug Delivery and Release Systems, College of Pharmaceutical Sciences, Zhejiang University; University of California Los Angeles; University of California, Los Angeles; University of California, Los Angeles; Tulane University; UCLA; University of California, Los Angeles

## Abstract

Embryos can achieve immune tolerance, yet the underlying mechanisms remain incompletely understood. Here, we demonstrate that pluripotent stem cells (PSCs), including embryonic stem cells (ESCs) and induced pluripotent stem cells (iPSCs), secrete extracellular vesicles (EVs) that markedly outperform mesenchymal stem cell (MSC)–derived EVs in suppressing pro-inflammatory cytokine secretion, inhibiting activated T-cell proliferation, and inducing regulatory T-cell (Treg) formation through CDK8 downregulation. Nuclear magnetic resonance (NMR) analysis reveals distinct molecular fingerprints of PSC EVs compared to those of MSC EVs. Moreover, comparative analyses show that PSC EVs contain unique proteins and microRNAs, such as the pluripotency-associated proteins ROR1 and CD133 and members of the miR-302 family, which are not found in MSC EVs, as determined by proteomic profiling and microRNA sequencing. Notably, the dynamic suspension culture of PSC aggregates significantly increases EV yield, offering a scalable and reproducible source superior to other cell sources. To evaluate their therapeutic potential, we employed an antigen-specific type 1 diabetes model and found that two local injections of iPSC EVs, particularly when delivered via a biomaterial scaffold, significantly enhanced diabetes-free survival. These treatments increased Treg populations in draining lymph nodes, induced systemic immunomodulation, and preserved β-cell mass from immune-mediated destruction. The immunomodulatory capability of PSC EVs suggests broad applications in treating autoimmune diseases and supporting stem cell-derived cell therapies by promoting immune tolerance. Their scalability, consistency, and superior therapeutic properties position PSC EVs as a compelling platform for next-generation immunotherapies and cell-based treatment strategies.

## Introduction

Extracellular vesicles (EVs) secreted by adult stem cells, particularly mesenchymal stem cells (MSCs), have attracted significant attention for their regenerative potential across a wide range of pathological conditions^1, 2, 3, 4, 5^. MSC-derived EVs (MSC EVs) have emerged as key mediators of intercellular communication^6^, capable of modulating immune responses and promoting tissue repair^7, 8, 9, 10, 11^. However, despite this promise, the clinical translation of MSC EVs is limited by ongoing challenges^12, 13, 14^, including donor variability and the finite proliferative capacity of MSCs^15^. Prolonged expansion of MSCs in culture inevitably leads to cellular senescence^16^, characterized by diminished function and regenerative potential, which also compromises the quality and therapeutic efficacy of their secreted EVs^17^. These limitations hinder the scalability and reproducibility of MSC EVs and present significant obstacles to their clinical application.

In contrast, pluripotent stem cells (PSCs), such as embryonic stem cells (ESCs) and induced pluripotent stem cells (iPSCs), exhibit unlimited expansion potential when cultured under optimal conditions^18^. This exceptional proliferative capacity enables large-scale production of both PSCs and their EVs, making them highly attractive for therapeutic development^19, 20^. Recent findings have demonstrated that PSC EVs not only stabilize the pluripotent state and prevent premature differentiation^21^ but also exhibit anti-senescent properties^19, 22, 23, 24, 25, 26^. However, their immunotherapeutic potential remains underexplored.

During early embryonic development, immune tolerance protects the semi-allogeneic fetus from maternal rejection. The trophoblast, which forms the placenta, is known to suppress maternal immune activation through the secretion of immunosuppressive factors and EVs^27^. In contrast, the immunomodulatory potential of the inner cell mass (ICM), the source of PSCs, remains largely unexplored. Motivated by these insights, we investigated whether ESCs and their artificially induced counterparts, iPSCs, secrete EVs that modulate immune cells and promote tolerance. We hypothesized that PSC EVs, reflecting the intrinsic properties of the ICM, are enriched with a unique set of immunoregulatory molecules that may surpass the well-established immunomodulatory properties of MSC EVs.

To investigate this, we developed a dynamic system for the large-scale production of EVs secreted by iPSCs and ESCs cultured as three-dimensional (3D) aggregates. We conducted a comprehensive analysis of the markers in these EVs, distinguishing them from those derived from adult stem cells. We established that the PSC EVs possess potent immunomodulatory properties. Not only were these properties evident *in vitro*, but localized delivery of iPSC EVs in a hydrogel that extended their release promoted systemic immune tolerance in an antigen-specific adoptive transfer model of type 1 diabetes (T1D).

## Results

### Characterization of iPSC and ESC EVs and dynamic culture expansion

**Figure 1:**
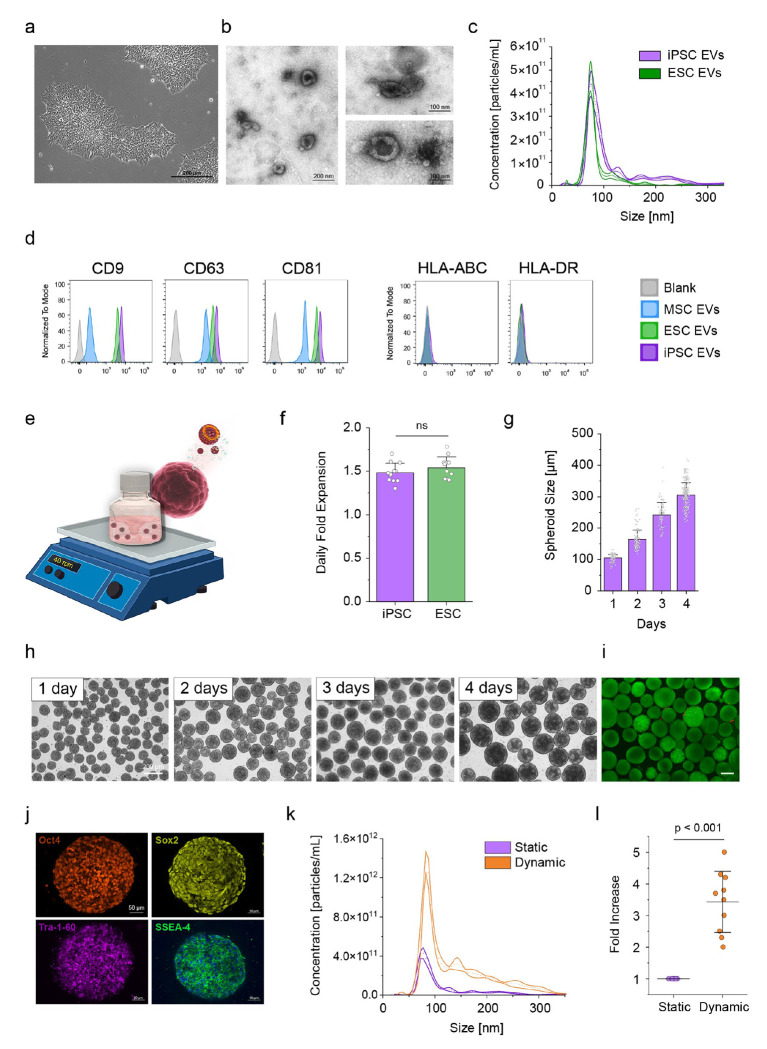
Characterization of iPSC and ESC EVs and dynamic culture expansion. **(a)** Morphology of iPSCs shown by phase-contrast microscopy (scale bar, 200 μm). **(b)** TEM images of EVs isolated from iPSC culture media, displaying typical EV morphology with a size around 100 nm. Scale bars: 200 nm (left) and 100 nm (right). **(c)** Nanoparticle tracking analysis (NTA) of iPSC and ESC EVs shows a comparable concentration of EVs, with the majority of particles around 100 nm in size. The dotted line represents the mean, while the solid lines indicate the standard deviation (SD); n = 5. **(d)** Flow cytometry analysis of EV surface markers (CD9, CD63, CD81) confirms their presence on iPSC, ESC, and MSC EVs. Minimal expression of immunogenicity markers HLA-ABC and HLA-DR in all EV samples, indicating a low immunogenic profile. **(e)** Schematic showing the dynamic culture system used for the formation of iPSC and ESC 3D aggregates and the production of EVs. PSCs are cultured as 3D aggregates in suspension under continuous agitation to maintain pluripotency and promote EV secretion. The illustration depicts the aggregation of stem cells and the release of nanoscale EVs into the culture medium. **(f)** Daily fold expansion of iPSC and ESC cells in dynamic culture shows no significant difference between the 2 cell types (ns = not significant). **(g)** Aggregate size increases progressively over 4 days of dynamic culture, as determined via microscopy. **(h)** Microscopy images showing spheroid morphology over 4 days of culture, demonstrating uniform spheroid growth (scale bar, 200 μm). **(i)** Live/dead staining of spheroids after 4 days in culture shows viable aggregates with minimal cell death (scale bar, 200 μm). **(j)** Immunofluorescence staining of iPSC spheroids after 4 days of dynamic culture reveals the expression of pluripotency markers (Oct4, Sox2, TRA-1-60, and SSEA-4), indicating the retention of pluripotent characteristics (scale bar, 50 μm). **(k)** NTA analysis of EVs produced under static and dynamic culture conditions shows increased EV production under dynamic conditions. The dotted line represents the mean, while the solid lines indicate the standard deviation (SD); n = 5. **(l)** Fold increase in EV production per million cells over 24 hours under static or dynamic culture conditions. Dynamic culture significantly enhances EV yield (p < 0.001). Dots represent individual data points, and lines indicate the mean ± standard deviation (SD).

To assess the potential of PSC EVs as scalable alternatives to MSC EVs, we first characterized the EVs produced by PSCs in both static and dynamic culture conditions. The morphology of iPSC colonies was confirmed via phase-contrast microscopy, which showed the compact, well-defined colony structure typical of PSCs (Fig. 1a). Isolated EVs from iPSC culture media, stained with uranyl acetate, exhibited the typical cup-shaped morphology under transmission electron microscopy (TEM), with sizes around 100 nm, consistent with small EV characteristics (Fig. 1b, Supplementary Fig. 1). Nanoparticle tracking analysis (NTA) further confirmed that EVs from iPSC as well as ESC cultures predominantly measured about 100 nm, and their concentrations were comparable, indicating similar EV production profiles between these 2 cell types (Fig. 1c). Additionally, the presence of classical EV surface markers—CD9, CD63, and CD81—on iPSC, ESC, and MSC EVs was confirmed by using a bead-based flow cytometry assay (Fig. 1d). In contrast, immunogenicity markers HLA-ABC and HLA-DR (major histocompatibility complex [MHC] class I and II molecules, respectively), which play a critical role in immune recognition by presenting antigens to CD8^+^ and CD4^+^ T cells and interacting with other immune cells, were minimally expressed on all tested EVs. This low expression suggests a reduced likelihood of immune activation and a favorable low immunogenic profile for iPSC and ESC as well as MSC EVs. Notably, the expression of these markers was tested on 6 iPSC lines, all of which showed similar results (Supplementary Fig. 2).

To enable scalable and efficient EV production, we utilized a dynamic culture system to grow PSCs as 3D aggregates (Fig. 1e). For smaller volumes (<100 mL), spheroids were cultured in flasks on an orbital shaker. The daily fold expansion rate for iPSCs and ESCs in the dynamic culture system showed no significant difference, indicating comparable cell growth between the 2 cell types (Fig. 1f). The dynamic culture system supported a progressive increase in spheroid size over a 4-day period (Fig. 1g), as evidenced by consistent growth observed via microscopy (Fig. 1h). Live/dead viability staining on day 4 confirmed minimal cell death within the spheroids, demonstrating the system’s capability to maintain high cell viability over time (Fig. 1i). Furthermore, immunofluorescence staining for pluripotency markers (Oct4, Sox2, Tra-1-60, and SSEA-4) verified that iPSCs retained their pluripotency in the 3D culture system (Fig. 1j).

EV production under dynamic culture conditions, measured after 24 hours by using NTA, was significantly higher than EV production under static conditions (Fig. 1k). Quantification of EV yield showed a statistically significant 3.4-fold increase in production per million cells during 24 hours of dynamic culture compared to static culture (Fig. 1l). These findings underscore the efficiency of dynamic culture for producing high yields of PSC EVs while preserving key attributes, such as EV markers and low immunogenicity (Supplementary Fig. 3).

### Distinct NMR fingerprint profile and multiomic markers of PSC EVs compared to those of MSC EVs

**Figure 2:**
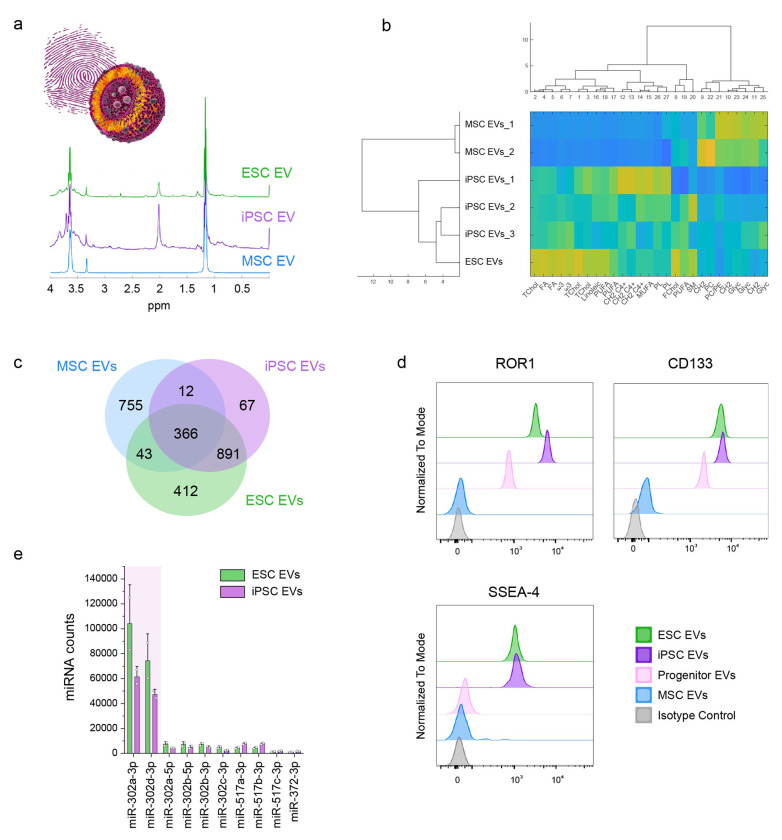
Comparative analysis of EVs derived from mesenchymal stem cells (MSCs), induced pluripotent stem cells (iPSCs), or embryonic stem cells (ESCs). **(a)** Schematic of an EV and its characteristic NMR spectral “fingerprint.“ The upper image illustrates the vesicle structure, and the spectra below show representative NMR profiles of lipids and metabolites found in ESC EVs (green), iPSC EVs (purple), and MSC EVs (blue). **(b)** NMR fingerprint comparison of MSC EVs, iPSC EVs, and ESC EVs. Dendrogram (top) and heatmap (bottom) display hierarchical clustering based on key spectral features across different EV groups. Distinct clustering indicates variation in EV lipid composition, with iPSC and ESC EVs showing a unique profile compared to the profile of MSC EVs. **(c)** Venn diagram illustrating the shared and unique protein profiles of MSC EVs, iPSC EVs, and ESC EVs. The MSC EV protein data were compiled from previously published studies ^28, 29, 30^ and the ExoCarta database ^31^, while iPSC and ESC EV proteins were measured by mass spectrometry in this study. A core set of 366 proteins is common to all three EV types, with 891 proteins specifically shared between iPSC EVs and ESC EVs. **(d)** Flow cytometry analysis of stem cell markers ROR1, CD133, and SSEA-4 across different EV populations. ESC EVs (green) and iPSC EVs (purple) express higher levels of these pluripotency markers compared to ESC-derived cardiac progenitor EVs (pink), MSC EVs (blue), and isotype controls (gray). **(e)** Counts of highly abundant miRNAs unique to iPSC and ESC EVs. ESC EVs (green) and iPSC EVs (purple) predominantly exhibit high expression levels of miR-302a-3p and miR-302d-3p, as determined by miRNA sequencing.

Given the complex nature of EVs, we performed a comprehensive analysis of the composition of EVs derived from iPSCs or ESCs to define a molecular fingerprint or a set of markers for quality control in EV production. Nuclear magnetic resonance (NMR) spectroscopy was employed as a powerful and non-destructive method to generate and analyze the NMR spectral fingerprints of EVs. This approach revealed distinct molecular signatures for EVs derived from different cell sources (Fig. 2a). Hierarchical clustering analysis based on NMR data further highlighted clear differences among EV types, with iPSC- and ESC-derived EVs clustering together as a separate group from MSC EVs (Fig. 2b, Supplementary Table 1). These findings underscore the unique chemical composition of PSC-derived EVs compared to MSC EVs.

To further characterize the molecular composition underlying these differences, we examined the primary contributors to the NMR spectral fingerprints. Our analysis revealed that the most prominent signals in the spectra originate primarily from lipids, which are key structural and functional components of EVs. Lipids produce sharp, well-defined peaks due to their simple chemical structures and uniform environments, such as hydrophobic fatty acid chains and polar head groups. In contrast, proteins and miRNAs experience diverse chemical environments caused by their complex 3D structures and varying interactions with other molecules, leading to weaker and broader peaks. These differences in signal characteristics make lipids the primary contributors to the NMR fingerprint of EVs, facilitating effective differentiation between EV types and providing means to assess variability across EV batches. Detailed lipid profiling further revealed that PSC EVs are enriched in cholesterol and polyunsaturated fatty acids (PUFAs), including omega-3, whereas MSC EVs contain higher levels of phosphatidylcholine (PC) and phosphatidylethanolamine (PE).

In addition to lipid composition, we investigated the protein content of iPSC- and ESC-derived EVs using mass spectrometry. Given that MSC EV protein composition can vary significantly depending on the cell source and other factors, we compared our results to previously published MSC EV datasets^28, 29, 30^ and the MSC EV proteins from ExoCarta database^31^; these datasets were consolidated into a single reference group for comparison (Supplementary Fig. 4). Our analysis of EV protein content identified proteins shared across different EV types and proteins unique to certain types (Fig. 2c). A core set of 366 proteins was found to be common to MSC, iPSC, and ESC EVs, representing conserved components across these vesicle types. However, significant differences in protein composition were observed. Notably, 755 proteins were unique to MSC EVs, reflecting their distinct molecular signature, while 891 proteins were shared exclusively between iPSC- and ESC-derived EVs and were absent in MSC EVs, underscoring the difference between PSC EVs and MSC EVs.

To explore the functional implications of these differences, we performed pathway enrichment analysis using MSigDB gene sets. Proteins shared across all EV types (ESC, iPSC, MSC) were enriched in pathways related to tissue remodeling, metabolism, and stress responses, including EMT, MYC targets, mTORC1 signaling, angiogenesis, glycolysis, and IL-2/STAT5 signaling (Supplementary Fig. 5). Proteins unique to iPSC/ESC EVs were primarily enriched in MYC and E2F targets, mTORC1 signaling, and the G2–M checkpoint, suggesting a biosynthetically active cargo that may support cell survival and transcriptional reprogramming. In contrast, MSC EV-specific proteins were enriched in coagulation, IL-6/JAK/STAT3 signaling, complement, EMT, and angiogenesis, consistent with their stromal origin and regenerative roles.

As specific examples of PSC EV markers, ROR1 (receptor tyrosine kinase–like orphan receptor 1) and CD133 were identified as proteins uniquely associated with PSC EVs. ROR1, highly expressed during embryonic development, plays roles in cell migration, differentiation, and tissue patterning, but is largely absent in adult tissues. CD133 is a well-established marker of stemness and self-renewal. To validate these markers, we performed flow cytometry of EVs using bead-based assays and compared the expression of ROR1, CD133, and the well-known stem cell marker SSEA-4 with their expression in MSC EVs and EVs from ESC-derived cardiac progenitors. As shown in Fig. 2d, iPSC and ESC EVs exhibited significantly higher expression of all three markers compared to MSC EVs, while EVs from differentiating cells showed reduced expression of these markers.

These findings demonstrate that, in addition to NMR, protein markers such as ROR1, CD133, and SSEA-4 can be used to effectively distinguish PSC EVs from MSC EVs and provide information on EVs’ cellular origin in the context of developmental status. The clear reduction of these markers in EVs from differentiated progenitor cells underscores their sensitivity as indicators of differentiation and changes in EV composition. Importantly, a comparison of EVs produced under static and dynamic culture conditions revealed no significant differences in the expression of these 3 markers (Supplementary Fig. 6), confirming that the dynamic culture process preserves the characteristic surface marker profiles of iPSC EVs. Protein marker analysis by flow cytometry, guided by mass spectrometry screening, is a valuable tool for quality control, allowing for the precise monitoring of EV production processes. By identifying deviations in marker expression, this approach can provide batch-to-batch consistency and detect potential contamination by EVs from differentiated cells, ensuring the functional integrity of EVs intended for therapeutic applications.

Finally, we performed microRNA sequencing of iPSC- and ESC-derived EVs and compared their miRNA profiles with those of MSC EVs from five different sources^32, 33, 34, 35, 36^ to identify miRNAs uniquely expressed in iPSC and ESC EVs (Supplementary Fig. 7). Our analysis revealed that iPSC and ESC EVs contain a diverse range of miRNAs, with miR-302a-3p and miR-302d-3p being the most highly expressed (Fig. 2e). These miRNAs belong to the miR-302 family, which plays a crucial role in maintaining pluripotency and promoting cellular reprogramming. Their enrichment in iPSC and ESC EVs reflects the molecular signature of PSCs and distinguishes these EVs from those derived from adult stem cells, which lack these miRNAs. The unique presence of miR-302a-3p and miR-302d-3p in iPSC- and ESC-derived EVs underscores their potential as robust biomarkers for identifying and characterizing EVs derived from PSCs, as well as for understanding the unique bioactive properties of these EVs.

### Enhanced *in vitro* immunomodulatory properties of PSC EVs compared to those of MSC EVs

**Figure 3:**
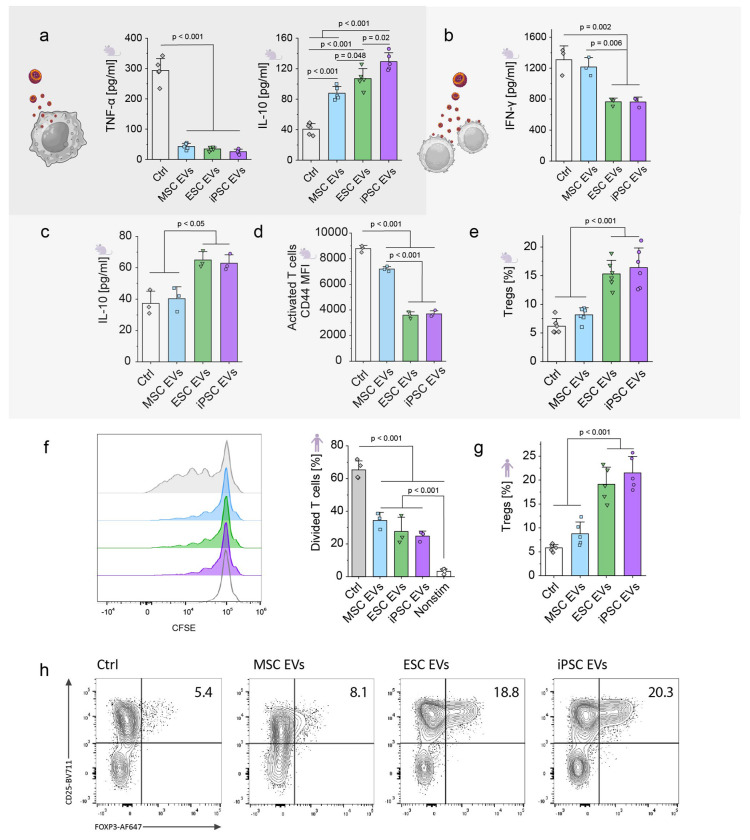
Immunomodulatory effects of EVs from MSCs, ESCs, and iPSCs on immune cells *in vitro*. **(a)** Cytokine secretion by mouse macrophages after the addition of EVs. EVs from MSCs (blue), ESCs (green), and iPSCs (purple) significantly reduced TNF-α production compared to a control group in which no EVs were added (Ctrl). All 3 EV types increased IL-10 secretion, with iPSC and ESC EVs having a greater effect than MSC EVs. **(b)** IFN-γ secretion by mouse T cells after EV addition. Compared to MSC EVs and the control group, ESC EVs and iPSC EVs reduced IFN-γ production, **(c)** IL-10 secretion by mouse T cells after EV addition. EVs from ESCs and iPSCs significantly increased IL-10 secretion, indicating their immunosuppressive potential. **(d)** Activation status of mouse T cells (CD44 MFI). EVs from ESCs and iPSCs reduced the activation of T cells, as shown by decreased CD44 mean fluorescence intensity (MFI), compared to MSC EVs and Ctrl. **(e)** Proportion of regulatory T cells (Tregs) within mouse CD4^+^ T cells. Compared to MSC EVs and the control group, ESC EVs and iPSC EVs significantly enhanced Treg formation. **(f)** Proliferation of human T cells after EV addition as determined by CFSE staining. Compared to the control group (gray), EVs from MSCs (blue), ESCs (green), and iPSCs (purple) suppressed the proliferation of human T cells. Nonstim (white) represents non-stimulated CD4^+^ T cells, which served as a baseline control for proliferation., **(g)** Proportion of Tregs in human CD4+ T cells. Compared to MSC EVs and the control group, ESC EVs and iPSC EVs significantly increased Treg formation. **(h)** Flow cytometry plots showing the proportion of Tregs (CD25+FoxP3+) in human CD4+ T cells after treatment with EVs. The percentage of Tregs increased substantially following the addition of ESC and iPSC EVs compared to MSC EVs and the control group.

Next, we evaluated the immunomodulatory effects of the isolated EVs *in vitro*. First, we compared the properties of human EVs derived from MSCs, ESCs, or iPSCs on mouse immune cells, including macrophages and T cells. Compared to a control group in which no EVs were added, EVs from all 3 sources decreased secretion of the proinflammatory cytokine TNF-α by mouse macrophages (Fig. 3a). However, ESC and iPSC EVs induced significantly higher IL-10 secretion than MSC EVs, suggesting stronger anti-inflammatory potential. In T cell culture, ESC and iPSC EVs, unlike MSC EVs, reduced IFN-γ secretion by mouse T cells (Fig. 3b) and significantly increased IL-10 secretion (Fig. 3c), whereas MSC EVs had minimal effect compared to the control group. The immunosuppressive properties of ESC and iPSC EVs were further demonstrated by their ability to modulate T-cell activation and promote regulatory T-cell (Treg) formation. ESC and iPSC EVs significantly reduced the activation of T cells, as indicated by the lower mean fluorescence intensity (MFI) of the CD44 activation marker compared to that of MSC EVs and the control group (Fig. 3d). Furthermore, ESC and iPSC EVs promoted the generation of Tregs at significantly higher levels than MSC EVs and the control group (Fig. 3e), indicating a strong capability to induce an immunoregulatory phenotype.

In human T-cell cultures, ESC and iPSC EVs also suppressed T-cell proliferation in comparison to the control group, as shown by the reduced percentage of divided cells stained with carboxyfluorescein succinimidyl ester (CFSE) (Fig. 3f). Moreover, ESC and iPSC EVs significantly increased the proportion of Tregs among human CD4^+^ T cells, further supporting their immunoregulatory potential (Fig. 3g). Flow cytometry plots provide a clear visualization of the increased Treg population (CD25^+^FoxP3^+^) following treatment with ESC and iPSC EVs, demonstrating their ability to induce an immunoregulatory phenotype, superior to that of MSC EVs and the control group (Fig. 3h).

### iPSC EVs reprogram immune cells in draining lymph nodes by downregulating CDK8

**Figure 4:**
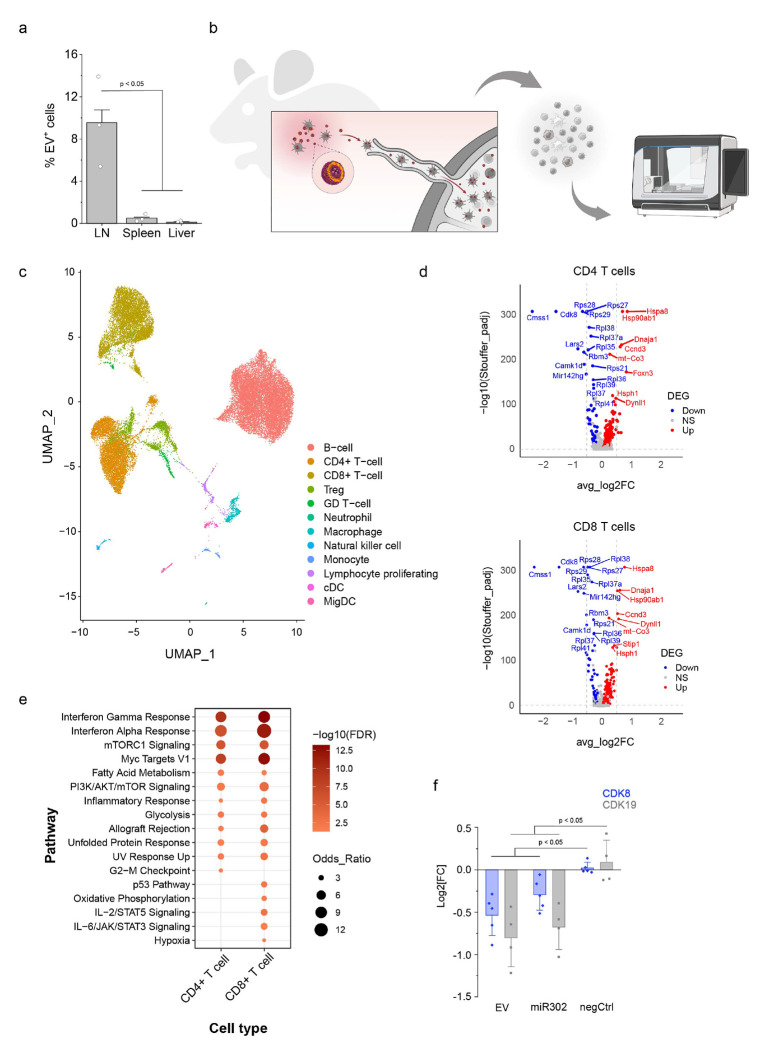
Single cell–sequencing analysis of cells in mouse lymph nodes following iPSC EV treatment. **(a)** Distribution of iPSC EVs in lymph nodes (LNs), spleen, and liver, as shown by the percentage of EV-positive cells in each organ 24 hours after subcutaneous EV injection. The majority of EV-positive cells are localized in the draining LNs. **(b)** Schematic representation of iPSC EV administration and subsequent single cell–sequencing analysis of immune cell populations in lymph nodes. **(c)** UMAP visualization of main immune cell populations identified in draining lymph nodes following iPSC EV treatment. Different cell types, including B cells, CD4^+^ T cells, CD8^+^ T cells, Tregs, macrophages, and dendritic cell subsets, are color-coded according to their identities. **(d)** Volcano plots showing DEGs in CD4^+^ T cells and CD8^+^ T cells following iPSC EV treatment. Notably, CDK8 expression is significantly downregulated in both T-cell populations, highlighting the modulatory impact of iPSC EVs on T cells. **(e)** Pathway enrichment analysis of CD4^+^ and CD8^+^ T cells following iPSC EV treatment. Dot plot showing significantly enriched Hallmark pathways in CD4^+^ and CD8^+^ T cells, based on differentially expressed genes from single-cell RNA-seq data. The color intensity represents the statistical significance of enrichment (−log_10_ FDR), while dot size indicates the odds ratio of enrichment. Key pathways include interferon responses, mTORC1 signaling, MYC targets, fatty acid metabolism, and IL-2/STAT5 signaling, suggesting broad transcriptional reprogramming involving cytokine signaling, metabolic adaptation, and immune regulation. **(f)** Relative expression levels of CDK8 (blue) and CDK19 (gray) in human CD4^+^ T cells treated with iPSC EVs, miR-302 mimics, or a negative control (negCtrl) after 3 days, as determined by qPCR. Expression levels are shown as log2 fold changes (Log2[FC]) relative to untreated controls. Both iPSC EVs and miR-302 mimics significantly downregulated CDK8 expression compared to the negative control group (*P* < 0.05). Data represent CD4^+^ T cells isolated from 4 to 5 individual donors.

Building on our *in vitro* findings, we next evaluated the *in vivo* effects of iPSC EVs. To assess their biodistribution, we covalently labeled iPSC EVs with a near-infrared (NIR) fluorescent dye and administered them subcutaneously into the neck region of C57BL/6J mice. After 24 hours, flow cytometry revealed that EV uptake was predominantly localized to the draining lymph nodes, with significantly fewer EV-positive cells detected in the spleen and liver (Fig. 4a).

To further investigate the impact of iPSC EVs on local immune cell populations, we performed single-cell RNA sequencing (scRNA-seq) on cells isolated from the draining lymph nodes five days post-injection (Fig. 4b). After preprocessing and quality control, we obtained transcriptomic profiles for 32,463 cells—15,905 from control mice and 16,558 from EV-treated mice. Dimensionality reduction using Uniform Manifold Approximation and Projection (UMAP) revealed distinct immune cell clusters (Fig. 4c), which were annotated based on canonical markers and aligned with a previously published immune cell atlas^37^. Major immune populations included B cells, multiple T-cell subsets, dendritic cells (both conventional and migratory), macrophages, monocytes, neutrophils, and NK cells (Fig. 4c, Supplementary Fig. 8).

Using UMAP overlays and differential gene expression analysis, we observed broad transcriptional shifts across several immune cell types following iPSC EV treatment (Supplementary Fig. 9). Notably, both CD4^+^ and CD8^+^ T cells exhibited significant changes in gene expression, including marked downregulation of cyclin-dependent kinase 8 (CDK8) (Fig. 4d). CDK8, along with its paralog CDK19, functions as a physiological suppressor of Foxp3 expression in conventional T cells (Tconv). Their inhibition is known to enhance STAT5 activation, thereby promoting Treg conversion even under inflammatory conditions^38^. These findings suggest that iPSC EVs may facilitate a regulatory T cell phenotype by suppressing CDK8 expression.

Pathway enrichment analysis of the CD4^+^ and CD8^+^ T cell subsets further supported this conclusion, revealing significant enrichment in hallmark gene sets related to cytokine signaling (e.g., interferon responses, IL-2/STAT5), metabolic reprogramming (e.g., mTORC1 signaling, MYC targets, fatty acid metabolism, PI3K/AKT/mTOR, glycolysis), and stress adaptation (e.g., unfolded protein response, p53 pathway) (Fig. 4e). These enrichments indicate broad transcriptional reprogramming consistent with immune modulation.

Consistent with this mechanism, we observed upregulation of IL-2/STAT5-responsive genes in CD8^+^ T cells (Supplementary Fig. 10). These genes are associated with cytokine responsiveness, survival, and a memory-like or regulatory phenotype, rather than effector activation. The absence of classical activation markers such as *Il2ra, Ifng, Gzmb*, and *Mki67* further suggests that iPSC EVs do not induce cytotoxic responses but instead promote a tolerogenic transcriptional program.

To validate these findings in human cells, we treated human CD4^+^ T cells with iPSC EVs and confirmed downregulation of *CDK8* and *CDK19* by qPCR (Fig. 4f). We then investigated whether this effect could be attributed to miR-302, the most abundant miRNA family in iPSC EVs. Treatment with miR-302 mimics similarly reduced *CDK8* and *CDK19* expression, suggesting that this miRNA may contribute to the immunomodulatory effects of iPSC EVs. While additional studies are needed to confirm this mechanism, our data highlight miR-302 as a key candidate mediating the suppression of CDK8/CDK19 and the induction of a regulatory T cell phenotype.

### A hyaluronic acid–Pluronic F127 hydrogel enables controlled delivery of iPSC EVs

**Figure 5:**
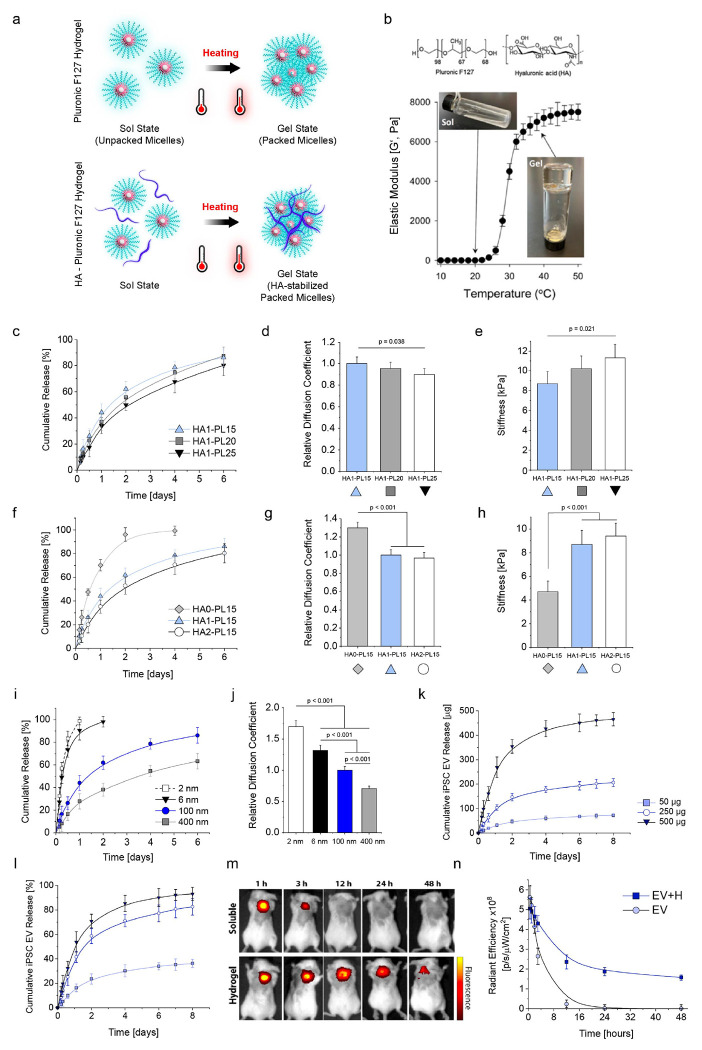
Development and characterization of a HA–Pluronic F127 hydrogel for controlled delivery of iPSC EVs. **(a)** Schematic of the thermal gelation process for a Pluronic F127 hydrogel and an HA–Pluronic F127 hydrogel. Upon heating, the solution state (sol) with unpacked micelles transitions to a gel state with packed micelles. In the HA–Pluronic F127 hydrogel, HA provides additional stabilization of the packed micelles in the gel state. **(b)** Temperature-dependent gelation of the HA–Pluronic F127 hydrogel. Elastic modulus (G′) measurements show a sharp increase as the temperature rises, indicating the transition from solution to gel state. Insets display the physical transformation from solution to gel. Chemical structures of Pluronic F127 and HA are shown above the graph. **(c)** Cumulative release of EVs from HA–Pluronic F127 hydrogel formulations with varying Pluronic F127 concentrations—PL15 corresponding to 15% (w/v); PL20, to 20%; and PL25, to 25% -over 6 days. Increasing Pluronic F127 concentration reduces the release rate. **(d)** Relative diffusion coefficients for EVs in HA–Pluronic F127 hydrogels with different Pluronic F127 concentrations (PL15, PL20, and PL25). Higher Pluronic F127 concentration reduces EV diffusion, as shown by a significant decrease in the diffusion coefficient. **(e)** Stiffness of HA–Pluronic F127 hydrogels with varying Pluronic F127 concentrations. Increased Pluronic F127 concentration results in greater hydrogel stiffness. **(f)** Cumulative release of EVs from HA–Pluronic F127 hydrogels with varying HA concentrations–HA0 corresponding to 0% (w/v); HA1, to 1%; and HA2, to 2%–over 6 days. Higher HA concentration decreases the release rate. **(g)** Relative diffusion coefficients for EVs in HA–Pluronic F127 hydrogels with different HA concentrations. Increased HA concentration significantly reduces EV diffusion.**(h)** Stiffness of HA–Pluronic F127 hydrogels with varying HA concentrations. Higher HA content results in a stiffer gel. **(i)** Cumulative release of nanoparticles of different sizes (2 nm, 6 nm, 100 nm, 400 nm) from HA–Pluronic F127 hydrogels over 6 days, demonstrating the impact of particle size on release dynamics. **(j)**Relative diffusion coefficients of nanoparticles in HA–Pluronic F127 hydrogels by size. Smaller particles exhibit higher diffusion rates than those of larger particles. **(k-l)** Cumulative release of iPSC EVs from the hydrogel over 8 days, measured for different EV doses (50 μg, 250 μg, and 500 μg), presented in absolute amounts (k) and as percentages (I). The results demonstrate dose-dependent release kinetics. **(m)** *In vivo* fluorescence imaging showing the retention of fluorescently labeled EVs in mice overtime. Soluble EVs are cleared rapidly, while EVs encapsulated in hydrogels show prolonged retention at the injection site, up to 48 hours.**(n)** Quantification of *in vivo*radiant efficiency, showing the extended retention of hydrogel-encapsulated EVs compared to soluble EVs over 48 hours.

Given that iPSC EVs accumulate in draining lymph nodes and modulate immune cell function, we sought to enhance their therapeutic potential by achieving controlled and prolonged release *in vivo*. To this end, we developed an injectable hydrogel composed of hyaluronic acid (HA) and Pluronic F127 that transitions from a solution to a gel state at body temperature. Figure 5a illustrates the thermal gelation process of this composite hydrogel. At low temperatures, Pluronic F127 and HA–Pluronic F127 hydrogels remain in a solution state with unpacked micelles. Upon heating, they transition to a gel state with packed micelles, where the presence of HA provides additional stabilization. Thermal gelation was confirmed by measuring the elastic modulus (G’) as a function of temperature. A sharp increase in G’ above 30 °C indicated the sol-to-gel transition (Fig. 5b), supported by visual confirmation of the transformation from liquid to gel.

The release kinetics of EVs from HA–Pluronic F127 hydrogels were influenced by the concentration of Pluronic F127. Higher concentrations of Pluronic F127 (PL20 and PL25, corresponding to 20 wt/v% and 25 wt/v%, respectively) reduced the cumulative release of EVs over 6 days (Fig. 5c). This effect corresponded to a significant decrease in the relative diffusion coefficient of EVs with increasing Pluronic F127 concentration (Fig. 5d). These changes were accompanied by increased hydrogel stiffness (Fig. 5e), indicating that Pluronic F127 concentration modulates both diffusion and mechanical properties.

Similarly, HA concentration played a critical role in regulating EV release. Hydrogels containing HA exhibited slower release rates (Fig. 5f), lower diffusion coefficients (Fig. 5g), and greater stiffness (Fig. 5h) compared to those without HA. However, there were no significant differences in stiffness or diffusion coefficient between hydrogels with 1% HA and hydrogels with 2% HA (w/v). Based on these findings, we selected the HA1-PL15 formulation, containing 1% HA and 15% Pluronic F127 (w/v), as the optimal choice due to its favorable stiffness, injectability, release profile, and gelation properties.

Particle size was another key factor influencing release dynamics. Smaller nanoparticles (2 nm and 6 nm) demonstrated higher cumulative release over 6 days compared to that of larger particles (100 nm and 400 nm) (Fig. 5i). This was consistent with the diffusion coefficients, which indicated that particle size directly impacts release rate (Fig. 5j). As EVs are approximately 100 nm in size, their release rate is expected to fall between that of smaller particles and 400-nm particles, favoring sustained release.

Cumulative release studies with iPSC EVs demonstrated dose-dependent release kinetics. Over an 8-day period, higher initial EV doses (500 μg) exhibited faster release, while lower doses (50 μg and 250 μg) showed slower release rates (Fig. 5k, l). This effect is attributed to the steeper concentration gradient at higher initial loading, which accelerates the release process. These findings suggest that adjusting the EV dose could provide an additional mechanism to fine-tune release profiles.

For *in vivo* studies, we selected an intermediate EV dose of 100 μg based on our preliminary studies demonstrating effective immune modulation at similar concentrations. To evaluate real-time EV release *in vivo*, we labeled iPSC EVs with the NIR dye and subcutaneously injected them, either encapsulated in the hydrogel or in soluble form, into the neck area of mice. *In vivo* imaging system (IVIS) imaging was used to track EV retention at the injection site. This fluorescence imaging revealed that EVs embedded in the hydrogel remained at the injection site for at least 48 hours, whereas soluble EVs were rapidly cleared within 3 hours (Fig. 5m). Quantitative analysis further confirmed that hydrogel embedding resulted in significantly higher radiant efficiency (Fig. 5n), highlighting its potential for prolonged local delivery of EVs.

Cytotoxicity testing was conducted to evaluate the safety of the hydrogel and iPSC EVs mixed with hydrogel. Key clinical chemistry and hematological parameters, including alanine aminotransferase (ALT), aspartate aminotransferase (AST), blood urea nitrogen (BUN), and creatinine, as well as red blood cell (RBC), white blood cell (WBC), platelet, and monocyte counts, were analyzed. ALT and AST levels indicated no liver damage, while BUN and creatinine confirmed normal kidney function (Supplementary Fig. 11). Hematological parameters were within normal ranges and indicated no pathological changes. These findings demonstrate that the hydrogel and iPSC EVs mixed with the hydrogel are non-toxic and biocompatible, supporting their safe use for prolonged local delivery of EVs in therapeutic applications.

### *In vivo* assessment of immunomodulatory properties of iPSC EVs using an adoptive-transfer T1D model

To evaluate the immunomodulatory effects of iPSC EVs *in vivo*, we employed an adoptive-transfer model of T1D. In this model, antigen-specific CD4^+^ T cells, which recognize a peptide expressed on pancreatic beta cells, were transferred from BDC2.5 transgenic mice into NOD-SCID mice to induce diabetes. Then, mice were treated with iPSC EVs alone, hydrogel alone, or iPSC EVs encapsulated in hydrogel, and diabetes progression was monitored over time.

**Figure 6:**
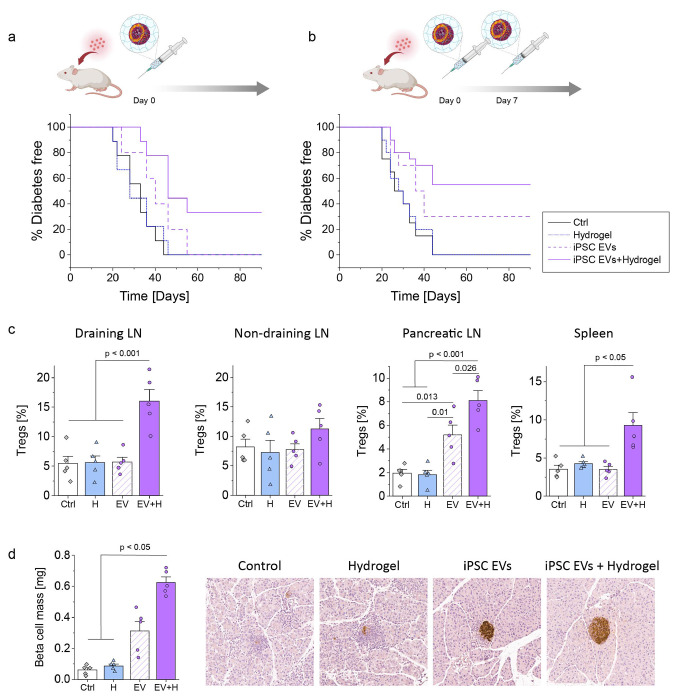
*In vivo* assessment of iPSC EVs using an adoptive-transfer type 1 diabetes mouse model. In this model, autoreactive CD4^+^ T cells, which target a specific peptide on pancreatic beta cells, were transferred from BDC2.5 transgenic mice into NOD-scid mice to induce diabetes. The immunomodulatory and protective effects of iPSC EVs were evaluated, both alone and in combination with a hydrogel delivery system. **(a)** Kaplan-Meier survival curve showing the percentage of diabetes-free mice after a single treatment (on day 0) in 4 groups: control (Ctrl), hydrogel alone (H), iPSC EVs alone (EV), and iPSC EVs combined with hydrogel (EV+H). iPSC EVs, especially in combination with hydrogel, significantly delayed diabetes onset compared to Ctrl and H. (n=10) **(b)** Kaplan-Meier survival curve showing percentage of diabetes-free mice after 2 doses (on day 0 and day 7) in the same groups. The EV+H group exhibited the greatest delay in diabetes onset, indicating enhanced therapeutic potential with repeated dosing. (n=10) **(c)** Percentage of Tregs in various lymphoid organs (draining lymph node [LN], non-draining LN, pancreatic LN, and spleen) measured by flow cytometry. Treatment with iPSC EVs in hydrogel (EV+H) resulted in a significant increase in Tregs, particularly in the draining and pancreatic LNs and the spleen, indicating localized and systemic immunosuppressive effects. **(d)** Quantification of the remaining beta cell mass in pancreatic tissue (left). The EV+H group maintained significantly greater beta cell mass compared to that of other groups, suggesting protection against autoimmune destruction. Representative histological images of pancreatic tissue from each treatment group, with beta islets stained for insulin (right). The Ctrl and H groups show minimal beta cell preservation, whereas iPSC EVs, especially with hydrogel (EV+H), preserved beta cell clusters.

Figure 6a illustrates the Kaplan-Meier curve showing the percentage of normoglycemic survival following a single treatment administered on day 0. Mice treated with iPSC EVs encapsulated in hydrogel displayed the highest percentage of normoglycemic survival compared to that of the control group, hydrogel alone, or iPSC EVs alone. While EVs alone conferred some degree of protection, encapsulating EVs in the hydrogel delivery system significantly enhanced their therapeutic efficacy.

When mice received 2 doses of treatment (on day 0 and day 7), Kaplan-Meier analysis demonstrated a further improvement in outcomes (Fig. 6b). At the end of the 90-day experiment, a greater percentage of diabetes-free mice were observed in the iPSC EV group (30%) and particularly in the group in which iPSC EVs were encapsulated in hydrogel (55%). This underscores the potential of repeated dosing to improve therapeutic efficacy and highlights the importance of sustained, localized EV delivery. The treatment of iPSC EVs encapsulated in hydrogel consistently outperformed all other groups, emphasizing the advantages of the hydrogel delivery system.

To investigate the underlying immunomodulatory mechanisms, we used flow cytometry to analyze the percentage of Tregs in lymph nodes and the spleen 10 days after the first EV injection. Treatment with iPSC EVs encapsulated in hydrogel significantly increased Treg populations, particularly in the draining lymph nodes, pancreatic lymph nodes, and spleen, compared to all other groups (Fig. 6c). Although EVs alone also increased Tregs in the pancreatic lymph nodes, the increase was less pronounced than in the group in which iPSC EVs were encapsulated in hydrogel. Hydrogel alone showed no significant immunomodulatory effects. These results suggest that the combination of iPSC EVs and hydrogel induces both localized and systemic immunosuppressive effects, which is critical for mitigating autoimmune responses.

Beta cell preservation was evaluated by visualizing insulin-stained beta cells and quantifying the remaining beta-cell mass in pancreatic tissue. Mice in the group where iPSC EVs were encapsulated in hydrogel retained a significantly greater beta-cell mass compared to the control and hydrogel groups (Fig. 6d). Histological analysis revealed substantial preservation of insulin-producing beta cell clusters in the group in which iPSC EVs were encapsulated in hydrogel, whereas the control and hydrogel groups exhibited almost complete beta cell destruction. EV treatment alone provided partial protection, but encapsulation in the hydrogel dramatically improved the outcomes.

These findings demonstrate that iPSC EVs, particularly when delivered via hydrogel, effectively suppress autoreactive T cells, enhance Treg induction, and preserve beta-cell mass in the antigen-specific adoptive-transfer model of T1D.

## Discussion

In this study, we demonstrate that EVs derived from PSCs, including ESCs and iPSCs, exhibit superior immunomodulatory properties compared to those of EVs from adult stem cells, such as MSCs. PSC EVs have unique molecular and functional characteristics, offering a promising avenue for scalable immunotherapeutic and regenerative applications.

The high proliferation rate of PSCs provides a distinct advantage for EV production. Compared to MSCs under static conditions, human iPSCs can produce up to 16 times more EVs^19^. While traditional adherent monolayer cultures are commonly used, suspension cultures have emerged as a scalable alternative. These allow PSCs to grow as floating aggregates, maintaining pluripotency without differentiation into embryoid bodies^39, 40^. Our results moreover show that PSCs cultured in dynamic suspension produce significantly more EVs while retaining key EV markers and stem cell-specific markers, supporting their clinical and industrial potential. Furthermore, the availability of cGMP-compliant iPSC lines ensures that PSC EV production can meet regulatory standards for therapeutic applications. Coupled with bioreactor-based suspension cultures, which are readily scalable, this provides a viable pathway for high-yield, clinically relevant EV manufacturing.

To date, the application of NMR spectroscopy in EV research has been limited. One notable example is the development of a μNMR microfluidic system for detecting circulating tumor EVs labeled with magnetic nanoparticles. This approach enabled the measurement of tumor EV concentration, providing a predictive metric for assessing therapy efficacy^41^. In our work, we employed label-free ^1^H-NMR spectroscopy for the characterization of EVs. Unlike optical methods such as Raman spectroscopy, which rely on vibrational modes and are prone to signal interference from overlapping peaks or fluorescence^42^, NMR provides high-resolution spectral data, enabling precise identification and quantification of metabolites and lipids. Furthermore, previous studies have demonstrated that NMR-based biofluid metabolomic profiles can reproducibly distinguish cancer patients from healthy controls^43^. Given that EV spectra contain fewer peaks compared to cellular spectra or complex biofluids, the clear identification of key components enhances both accuracy and reproducibility. Additionally, major lipid peaks could be quantified using integrated peak ratios relative to DSS (4,4-dimethyl-4-silapentane-1-sulfonic acid) as an internal standard. This approach could help establish a standardized method for evaluating lipid composition, detecting batch-to-batch variability, and improving reproducibility in EV production. These advantages position NMR as a powerful tool for EV characterization and quality assessment, making it particularly well suited for batch consistency testing in EV production.

Proteomic analysis further distinguished PSC EVs from MSC EVs, identifying 891 proteins uniquely present in iPSC and ESC EVs, including the surface markers ROR1 and CD133. Flow cytometry confirmed that both markers, along with the pluripotency-associated SSEA-4, were significantly more abundant in PSC EVs. Importantly, their expression was maintained under dynamic suspension culture conditions, highlighting the scalability and reproducibility of this EV production method.

ROR1 is highly expressed during embryogenesis and plays roles in cell migration, differentiation, and tissue patterning, but is largely absent in adult tissues^44^. CD133 (PROM1), a well-established marker of stem and progenitor cells, is involved in key cellular functions such as proliferation and often localizes to cholesterol-rich membrane microdomains^45^. Notably, our NMR analysis revealed that PSC EVs are enriched in cholesterol, suggesting a possible link between CD133 expression and the lipid composition of these vesicles.

According to miRNA sequencing, PSC EVs are highly enriched in the miR-302 family, particularly miR-302a-3p and miR-302d-3p, which are key regulators of pluripotency and reprogramming^46, 47, 48^. These miRNAs, tightly regulated by core pluripotency transcription factors (Oct4, Sox2, Nanog)^49^, are known to influence self-renewal, differentiation, and cellular reprogramming. Furthermore, the miR-302 family has demonstrated regenerative properties in respiratory bacterial infections by modulating inflammatory responses and promoting the proliferation of alveolar epithelial progenitor cells^50, 51^.

Our findings demonstrate that PSC EVs, enriched in miR-302 miRNAs and biosynthetically active protein cargo, possess potent immunomodulatory properties. While both PSC EVs and MSC EVs reduced TNF-α secretion by macrophages *in vitro*, PSC EVs significantly increased IL-10 production, highlighting their stronger anti-inflammatory potential. In T-cell cultures, PSC EVs more effectively reduced activation, suppressed proliferation, and increased Treg populations compared to MSC EVs, in both mouse and human systems. This enhanced ability to modulate pro- and anti-inflammatory signaling underscores the potential of PSC EVs to promote immune tolerance.

When injected subcutaneously into a mouse model, iPSC EVs preferentially localized to draining lymph nodes and influenced immune cell populations. These findings are consistent with other *in vivo* tracking studies of EVs using radiolabeling, which have demonstrated their preferential uptake by lymph nodes and the spleen^52^. Single-cell RNA sequencing revealed significant transcriptional changes in multiple immune cell types, highlighting changes in CD4^+^ and CD8^+^ T cells within the lymph nodes. Among the affected genes, the downregulation of CDK8 was particularly notable. CDK8 and its closely related paralog CDK19 are components of the transcriptional Mediator complex. In pluripotent stem cells, CDK8/19 inhibition enhances Mediator-driven recruitment of RNA polymerase II to promoters and enhancers, stabilizing naïve pluripotency^53^.

Beyond their role in maintaining pluripotency, CDK8 and CDK19 are known physiological repressors of *Foxp3* expression in activated T cells, and their inhibition has been shown to enhance STAT5 activation^38^. Previous studies have demonstrated that chemical inhibition or genetic knockdown of CDK8/19 induces *Foxp3* expression in both naïve and antigen-stimulated T cells, thereby promoting Treg differentiation even in inflammatory environments^38^. In our study, the downregulation of *Cdk8* in CD4^+^ and CD8^+^ T cells following iPSC EV treatment may represent a key mechanism by which PSC EVs promote Treg conversion.

Consistent with this mechanism, the IL-2/STAT5 signaling pathway was among the significantly enriched gene sets in CD8^+^ T cells after iPSC EV administration. One contributing factor may be the miR-302 family, which is highly enriched in PSC EVs and has been shown to regulate CDK8 expression. In addition, the EV proteome itself may support this effect: PSC EVs were enriched in proteins associated with MYC and mTORC1 signaling, E2F targets, and IL-2/STAT5 pathway components—biosynthetic and transcriptional programs that may further reinforce the immune-modulatory activity of PSC EVs.

The immunomodulatory properties of iPSC EVs were further evaluated using an antigen-specific T1D model. In this adoptive transfer model, CD4^+^ T cells specific for a beta cell antigen were transferred from BDC2.5 transgenic mice to NOD-scid mice, inducing diabetes. Hydrogel encapsulation enabled controlled, localized EV release, ensuring prolonged bioavailability and sustained immune modulation. Kaplan-Meier analysis demonstrated that a single administration of EVs encapsulated in hydrogel provided superior protection compared to that of EVs or hydrogel alone. Repeated dosing further improved outcomes, with 55% of mice remaining diabetes-free at the end of the experiment.

These findings align with previous observations that prolonged biomaterial-supported EV presentation and controlled release enhance therapeutic outcomes^2^. Sustained delivery systems like hydrogels protect EVs from rapid clearance, allowing gradual release and reducing the need for frequent dosing. Despite the localized administration in the neck region, systemic effects were observed, including increased Treg populations in the pancreatic lymph nodes and spleen. This suggests that PSC EVs delivered via hydrogel exert both local and systemic immunosuppressive effects.

Notably, systemic immune modulation from locally delivered EVs has also been observed in other contexts. For example, an inhalable therapeutic cancer vaccine based on EVs containing IL-12 mRNA induced systemic immune responses, demonstrating the potential of EV-based immunotherapeutics to modulate distant immune cells without causing severe side effects^54^.

The ability of PSC EVs to induce systemic immune tolerance following localized delivery represents a significant advancement in cell-free immunotherapies. By fostering a regulatory T-cell phenotype, these vesicles hold promise as potential treatments for autoimmune diseases such as rheumatoid arthritis, multiple sclerosis, and inflammatory bowel disease, where Treg dysfunction plays a central role. Additionally, PSC EVs could aid in promoting transplant tolerance by mitigating alloimmune responses, further expanding their therapeutic relevance.

In summary, our study underscores the scalability, reproducibility, and therapeutic potential of PSC-derived EVs. Dynamic suspension culture enables high-yield EV production while maintaining EV quality, and advanced characterization techniques like NMR facilitate rapid phenotyping, distinguishing PSC EVs from other EV types and ensuring batch-to-batch quality control. The unique cargo of PSC EVs, particularly miRNAs such as members of the miR-302 family, and their superior immunomodulatory properties distinguish them from other therapeutic EVs documented to date. Additionally, the development of a hydrogel-based sustained delivery system enhances their clinical applicability by ensuring prolonged bioavailability and effective systemic immune modulation. Collectively, these findings position PSC EVs as a promising tool for advancing next-generation immunotherapies and cell-free regenerative therapies, with potential applications in autoimmune diseases, transplantation, and immune tolerance induction.

## Methods

### Cells

Six iPSC cell lines were utilized in this study, including NH50191 iPSCs obtained from the NINDS Human Cell and Data Repository, powered by Sampled. Additionally, a B2M/CIITA double-knockout (HLA-I/II knockout) iPSC line was used, as described in a previous study^55^. Further iPSC lines included hIPSC 2 and hIPSC 23, along with BSCRC 23F1i3 from the UCLA core facility and iPS DF6-9-9T.B from WiCell. The ESC line H9 was also sourced from WiCell. For static cultures, both iPSC and ESC cells were plated on cell culture plates pre-coated with vitronectin (Stem Cell Technologies) and maintained in mTeSR Plus medium (Stem Cell Technologies). To enhance cell survival during the initial plating, Y-27632 (Cayman Chemical) was added at a final concentration of 10 μM for the first 24 hours. Medium changes were performed every 24 hours throughout the culture period. Human MSCs were obtained from 2 sources: the Texas A&M Health Science Center College of Medicine and ATCC (PCS-500-012). MSCs were cultured in RoosterNourish-MSC medium (PCS-500-041) provided by RoosterBio.

### Dynamic culture of iPSC and ESCs

For dynamic culture, iPSC and ESC cells were dissociated into small aggregates using ReLeSR (Stem Cell Technologies) and seeded into Nalgene^™^ Rapid-Flow Sterile Filter Storage Bottles (455-0250) (ThermoFisher Scientific). The culture medium was supplemented with Y-27632 at a final concentration of 10 μM. The bottles were placed on an orbital shaker, with a shaking speed of 55 rpm for 30-mL cultures or 65 rpm for 60-mL cultures. After 24 hours, the medium was replaced with TeSR-E8 3D medium (Stem Cell Technologies) to initiate EV collection. Cell aggregates were maintained in culture for up to 4 days, ensuring that the spheroid size did not exceed 500 μm in diameter. Regular assessments of aggregate viability were conducted using Trypan Blue staining or Live/Dead staining. Following the culture period, the cell aggregates were gently disintegrated into smaller clusters by using Gentle Cell Dissociation Reagent and passed through 37-μm Reversible Strainers (both from Stem Cell Technologies) before being re-seeded for subsequent dynamic culture cycles.

Immunofluorescence staining was performed as previously described (51). The primary antibodies used were anti-SSEA-4 (Santa Cruz Biotechnology, sc-21704), anti-Oct3/4 (Santa Cruz Biotechnology, sc-5279), anti-Sox2 (Merck Millipore, AB5603), and anti-TRA-1-60 (clone TRA-1-60, Sigma-Aldrich, MAB4360). Imaging was conducted with a Zeiss Axio Observer Z1 inverted fluorescence microscope, and the data were analyzed with ImageJ software.

### EV isolation

For EV collection, iPSC and ESC cultures were maintained in TeSR-E8 or TeSR-E8 3D medium (Stem Cell Technologies) for static and dynamic cultures, respectively. MSC EV collection was performed using RoosterCollect^™^-EV medium (M2001) from RoosterBio. After conditioning by the cells, the medium was centrifuged at 2000 × g for 10 minutes to eliminate large particles and debris. The supernatant was either processed immediately or frozen at −80 °C for later use. EVs were isolated from the cell-conditioned medium (CCM) through sequential filtration and size exclusion chromatography (SEC). First, the CCM was filtered through a 0.22-μm filter to eliminate larger EVs and remaining debris. The filtered CCM was then concentrated 100-fold using Centricon Plus-70 centrifugal filters (Ultracel PL-10, 10,000 NMWL, MilliporeSigma). The concentrated sample was loaded onto a pre-equilibrated qEVoriginal SEC column (IZON Science) to separate EVs from smaller protein contaminants. Fractions containing small EVs were identified, pooled, and concentrated 5-fold using Amicon Ultra 2-mL centrifugal filters (Ultracel-10K, 10,000 NMWL, MilliporeSigma). The protein concentration of the isolated EVs was quantified using the Micro BCA^™^ Protein Assay Kit (Thermo Scientific) according to the manufacturer’s protocol. The final concentrated EV preparations were stored at −80 °C until further use. TEM of EVs was performed as previously described^56^. The size distribution and concentration of EVs were determined by using a NanoSight NS300 system (Malvern Instruments). Prior to analysis, EV samples were diluted in 0.02 μm-filtered PBS to ensure optimal particle concentration. For each sample, five 30-second videos were captured with the camera set to level 14. The resulting video data were subsequently processed using NTA software version 3.4 to obtain particle size and concentration profiles.

### Evaluation of EV surface markers

To assess EV surface markers, the MACSPlex Exosome Kit (130-122-209, Miltenyi Biotec) was employed, following the manufacturer’s guidelines. EV samples were incubated with capture beads, which specifically bind to surface proteins of interest, such as ROR1, CD133, and SSEA-4. After binding, the bead-EV complexes were labeled using fluorescence-conjugated antibodies targeting CD9, CD63, and CD81. These labeled complexes were then subjected to flow cytometry to detect and quantify the fluorescence signals, enabling marker profiling. The analysis was performed using a ThermoFisher Attune NxT Flow Cytometer, and data interpretation was carried out with FlowJo v10 software.

### NMR analysis of EVs

The NMR buffer was prepared by dissolving 0.9% (w/v) NaCl and 0.001 mM sodium trimethylsilyl propane sulfonate (DSS, TCI Chemicals) in a solution containing 90% (v/v) D_2_O and 10% (v/v) H_2_O. EV samples were standardized to 100 μg/mL, and all spectra were acquired at this concentration. EVs were resuspended in the buffer and transferred to 5-mm NMR tubes for analysis. Proton (^1^H) NMR free induction decays (FIDs) were acquired at 298 K using a Bruker DRX-500 spectrometer operating at 500 MHz. Spectra were collected for each EV sample, averaging 32 FIDs after a set of dummy scans. Acquisition parameters included a spectral width of 6,009.62 Hz, 8k data points, and an acquisition time of 0.7 s per scan. Pulse delays ranged from 9 to 14 μs, and water signal suppression was achieved using the Bruker zgesgp pulse sequence. Raw NMR data were Fourier transformed and processed for automatic phase and baseline correction using TopSpin (version 3.6.3). The DSS signal was used as an internal chemical shift reference, calibrated to 0 ppm. Data analysis was performed in MATLAB (version 2022b) using the Edison Lab Metabolomics package. For each spectrum, the region corresponding to water peaks (4.2–5 ppm) was manually set to zero. Spectra were normalized to their total area, with each sample scaled to a total spectral area of 1.0. Lipid peaks characteristic of EVs were identified based on literature and compared with prominent peaks in the acquired spectra. Peaks with a signal-to-noise ratio (SNR) below 2 were excluded. Twenty-eight peaks were identified, and a spectral bin with a width of 0.01 ppm was centered on each assigned peak’s chemical shift, resulting in 28 bins per spectrum. Each bin was labeled according to the corresponding lipid peak. Signal integrals within each bin were computed, producing a data matrix of 28 bins × 6 samples. The mean and standard deviation for each bin were calculated across six samples, and mean values were subtracted from corresponding matrix entries to create a zero-mean matrix. Each element was then normalized by its respective standard deviation, yielding a matrix with zero mean and unit variance.

Hierarchical clustering analysis was performed on the standardized 28 × 6 matrix using MATLAB. The Ward algorithm and Euclidean distance metric were employed to iteratively link clusters, identifying overall clustering patterns among samples based on normalized spectral data. No additional statistical filtering was applied before clustering, ensuring an unbiased comparison of peaks regardless of statistical significance.

#### Proteomics of iPSC and ESC EV samples

EV peptide preparation was adapted from the single-pot, solid phase–enhanced sample preparation (SP3) protocol^57^. Briefly, 10 μg of EV sample was mixed with a reconstitution solution containing pH 8 HEPES (Sigma-Aldrich, cat. no. H3375), SDS (Fisher Bioreagent, cat. no. BP166-500), Triton X-100 (Sigma-Aldrich, cat. no. X100-500ML), NP-40 (EMD Millipore, cat. no. 492016-100ML), Tween 20 (Promega, cat. no. H5152), sodium deoxycholate (Sigma-Aldrich, cat. no. 30970-25G), EDTA (Fisher Bioreagent, cat. no. BP120-500), 200 mM NaCl (Fisher Chemical, cat. no. S271-1), glycerol (Fisher Bioreagent, cat. no. BP229-4), complete protease inhibitor (Sigma-Aldrich, cat. no. 04693132001), DTT (Sigma-Aldrich, cat. no. D0632-10G), and HPLC-grade water (Fisher Chemical, cat. no. AAB-W6-4). The solution was incubated for 30 minutes at 60 °C with gentle agitation at 1000 rpm. IAA (Sigma-Aldrich, cat. no. I6125-10G) was then added, and the mixture was incubated for 30 minutes at room temperature in the dark. DTT was subsequently added to quench alkylation for 15 minutes at room temperature.

The samples were sonicated for 10 cycles of 30 seconds ON/OFF. SP3 beads, prepared from a 1:1 mixture of hydrophilic and hydrophobic Sera-Mag carboxylate-modified magnetic beads (Cytiva, cat. no. 24152105050250 and 44152105050250), were added and gently mixed. Ethanol (Thermo Scientific, cat. no. 615090010) was added, and the mixture was incubated at 24 °C for 15 minutes at 1000 rpm to induce protein binding to the beads. The samples were placed on a magnetic rack to remove the supernatant, and the beads were washed twice with 80% ethanol followed by a rinse with acetonitrile. Trypsin/Lys-C (Promega, cat. no. V5073) was added at an enzyme-to-protein ratio of 1:25 in pH 8 ammonium bicarbonate solution. The beads were sonicated for 30 seconds to disperse and mix them followed by incubation at 37 °C for 18 hours at 1000 rpm for digestion. Following digestion, the samples were centrifuged at 20,000 × g for 1 minute, placed on a magnetic rack, and the supernatant was collected for LC-MS/MS analysis. LC-MS/MS Analysis: LC-MS/MS was performed with an Orbitrap Exploris 480 Mass Spectrometer (Thermo Fisher Scientific) coupled to a Vanquish Neo system (Thermo Fisher Scientific). Peptides were loaded onto a 2-μm C18 75 μm × 500 mm EASY-Spray^™^ PepMap^™^ Neo UHPLC column (Thermo Scientific, cat. no. ES75500PN). Separation was achieved at a flow rate of 0.2 μL/min by using a gradient of buffer B (80% ACN and 0.1% formic acid), starting from 5% to 23% over 101 minutes, increasing to 40% over 35 minutes, followed by a rise to 99% over 17 minutes, and a return to 5% buffer over 20 minutes. The total run time was 163.1 minutes, and the column temperature was maintained at 35 °C. Data-dependent acquisition (DDA) was performed with a full scan range of 350–1200 m/z at a resolution of 60,000, a normalized AGC target of 300%, and an RF lens of 40%. MS/MS spectra of the top 20 most abundant precursors (charge state 2–6) were acquired within an isolation window of 1.4 m/z at a resolution of 15,000, with a normalized AGC target of 50% and HCD collision energy of 30%. MS Data Analysis: DDA raw files were analyzed using Proteome Discoverer software (Thermo Fisher Scientific) with a label-free quantification workflow. Analysis was conducted according to a FASTA file containing MaxQuant contamination sequences and reviewed for Homo sapiens protein sequences, using default settings. Mass spectrometry data was processed and normalized with the Thermo Proteome Discoverer software (v. 2.4). The normalization mode selected was the total peptide amount, which sums the peptide group abundances for each sample and determines the maximum sum for all files. The normalization factor is the factor of the sum of the sample and the maximum sum in all files. Proteins with missing values in more than half of the samples in each group were removed. Missing data imputation was performed using the Random Forest imputation algorithm from the MissForest R package^58^. Highly abundant proteins in both ESC and iPSC EVs were defined as having a Thermo Proteome Discoverer classification of “Highly Abundant” in every replicate for each cell line.

### ESC and iPSC EV protein comparison with MSC EV proteins

MSC EV proteins were curated from previously published studies^28, 29, 30^ and the ExoCarta database^31^. As the number of reported proteins and methods for quantification of reads differed across studies, highly abundant MSC EV proteins were defined as the top 25% most abundant proteins detected across all samples^28, 29^, the top 10% most abundant proteins with expression >0^30^, and the MSC EV proteins curated in the Exocarta resource. MSC EV proteins identified by these studies were compared against the ESC and iPSC EV proteins to determine consistent and divergent trends. Pathway enrichment analysis was performed using EnrichR^59^ with the MSigDB Hallmark 2020 gene set collection. Analyses were conducted separately for proteins shared among all three EV types (ESC, iPSC, MSC), proteins shared only between ESC and iPSC, and proteins unique to MSC. Pathways with adjusted p-values < 0.05 were considered significantly enriched.

### miRNA Sequencing in iPSC and ESC EVs

miRNA sequencing of iPSC and ESC EVs was conducted by System Biosciences. Raw fastq data files generated using the Illumina NextSeq 5000 platform were processed by first trimming adaptors from the 3′ end of the reads using Trim Galore, which integrates Cutadapt and FastQC tools. The UMI-tools “Extract” function was applied to paired-end reads to extract UMI barcodes from the fastq files. A specific adaptor sequence (“AACTGTAGGCACCATCAAT”) was then removed from the reads using Trim Galore. Processed reads were mapped to the human hg38 reference genome by using the Extra-Cellular RNA Processing Toolkit (exceRpt). The exceRpt pipeline includes filtering and quality control steps to remove contaminants before mapping reads to endogenous sequence databases, including the hg38 genome, miRNAs from miRbase^60^, tRNAs from gtRNAdb^61^, piRNAs from piRNABank^62^, long RNAs from GENCODE^63^, and circRNAs from circBase^64^.

Highly abundant miRNAs in ESC and iPSC EVs were defined as the top 25% of miRNAs with >100 reads across samples. MSC EV miRNAs were curated from previously published studies^32, 33, 34, 35, 36^. Due to differences in profiling methods and reported values across these studies, including varying numbers of miRNAs captured, different quantification methods, and sequencing platforms, highly abundant MSC EV miRNAs were defined using different criteria. For Reis et al.^32^ and De Luca et al.^33^, the top 25% of miRNAs by read abundance were considered. Kaur et al.^34^ defined highly abundant miRNAs as the top 25% of miRNAs with >100 reads across samples, while Shafti et al.^35^ used the top 25% of miRNAs with <130 CTcorr values across samples. In the study by Liu et al.^36^, highly abundant miRNAs were defined as those with an average expression >1.

### *In vitro* experiment with immune cells

Mouse monocytes were isolated from the femurs of mice. After centrifugation at 1000 × g for 10 minutes at room temperature, the supernatant was discarded, and the cell pellet was resuspended in 25 mL of DMEM supplemented with 10% FBS. The cells were incubated at 37 °C for 6 hours. Following incubation, non-adherent cells were collected, pelleted, and resuspended in complete RPMI 1640 medium (RPMI 1640 supplemented with 10% heat-inactivated FBS, 1% penicillin-streptomycin (10,000 U/mL), 1 mM sodium pyruvate, 2 mM L-glutamine, 10 mM HEPES buffer, and 50 μM 2-mercaptoethanol) containing 50 ng/mL M-CSF to facilitate macrophage differentiation. On day 3, the medium was replaced with fresh complete RPMI containing 20 ng/mL M-CSF, with the same step repeated on day 5. By day 7, differentiated macrophages were detached using a cell scraper and seeded for downstream experiments. iPSC, ESC, or MSC EVs were added to the macrophage cultures at a final concentration of 10 μg/mL. After 24 hours of incubation, the culture medium was collected and analyzed for TNF-α and IL-10 levels via ELISA (Eve Technologies). Culture media containing EVs, but not exposed to cells, were used as negative controls.

Mouse T cells were isolated from spleens and lymph nodes using the EasySep Mouse T-Cell Isolation Kit (19851) or CD4^+^ T-Cell Isolation Kit (19852, Stem Cell Technologies) and cultured in complete RPMI 1640 medium. T-cell activation was performed on plates pre-coated with anti-CD3 (clone 2C11, Bio X Cell, BE0001-1) at 10 μg/mL and 2 μg/mL soluble anti-CD28 (clone 37.51, Bio X Cell, BE0015-1). IL-2 (25 U/mL, BRB Preclinical Repository, National Cancer Institute) and EVs (iPSC, ESC, or MSC, 10 μg/mL) were added, and cells were cultured for 3 days. Culture media were collected for IFN-γ and IL-10 analysis via ELISA (Eve Technologies), with EV-only media as negative controls. For proliferation analysis, cells were stained with Alexa Fluor 488 anti-mouse CD3 Antibody (100210) and Brilliant Violet 711 anti-mouse CD44 Antibody (103057), and viability was assessed using the Zombie NIR Fixable Viability Kit (423105, BioLegend). Treg formation was evaluated after 5 days by staining for CD4 (Brilliant Violet 605 anti-mouse CD4 Antibody, 100548), CD25 (Brilliant Violet 785 anti-mouse CD25 Antibody, 102051), and Foxp3 (PE anti-mouse FOXP3 Antibody, 126404) after fixation and permeabilization using the Foxp3 Fix/Perm Buffer Set (BioLegend). Data were acquired on a ThermoFisher Attune NxT Flow Cytometer and analyzed using FlowJo v10 software.

Human T cells were isolated from PBMCs obtained from the UCLA/CFAR Virology Core Laboratory using EasySep Human T-Cell Isolation Kit (17951) or CD4^+^ T-cell Isolation Kit (17952, Stem Cell Technologies). Activation was performed by using Dynabeads Human T-Activator CD3/CD28 (Gibco, 11161D) or by pre-coating plates with anti-human CD3 (1 μg/mL, Bio X Cell, BE0231) and adding 5 μg/mL anti-human CD28 (Bio X Cell, BE0248) to the medium. For proliferation assays, T cells were stained with 5 μM CFSE (Invitrogen, C34554) for 5 minutes at room temperature in the dark, washed, and cultured with EVs and IL-2 (25 U/mL, TECIN^™^ Teceleukin). Treg formation was assessed after 5 days by staining for CD45 (Alexa Fluor 488 anti-human CD45 Antibody, 160306), CD4 (Brilliant Violet 605 anti-human CD4 Antibody, 300555), CD25 (Brilliant Violet 711 anti-human CD25 Antibody, 356137), and FOXP3 (Alexa Fluor 647 anti-human FOXP3 Antibody, 320013, BioLegend).

### *In vivo* tracking of EVs

EVs were covalently conjugated to Flamma 675NA NHS ester dye by incubating them overnight at 4 °C with 1 mg/mL dye solution under continuous shaking. Unbound dye was quenched by incubating the mixture with 1 M Tris-HCl (pH 8) for 1 hour at 37 °C. Labeled EVs were then purified by using SEC as previously described. A total of 100 μg of labeled iPSC EVs was injected subcutaneously into wild-type C57BL/6 mice. After 24 hours, draining lymph nodes, spleen, and liver were harvested and dissociated into single-cell suspensions. EV uptake by cells from these tissues was evaluated by flow cytometry.

### Single-cell sequencing (scRNAseq) of immune cells

Wild-type C57BL/6 mice were treated with a total of 100 μg of iPSC EVs via subcutaneous injection, while control animals received PBS injections. After 5 days, draining lymph nodes were harvested, placed in cold, sterile PBS supplemented with 2% FBS to preserve cell viability. The lymph nodes were mechanically disrupted and passed through a 70 μm cell strainer to obtain a single-cell suspension. The filter was rinsed with additional media to maximize cell recovery. The resulting cell suspension was stained with trypan blue and counted using a hemocytometer. Only samples with high viability (≥80%) were selected for sequencing. No prior cell enrichment was performed. An aliquot of the prepared suspension was examined under a microscope to confirm cell integrity and the absence of clustering artifacts.

Following the isolation of single-cell suspensions (two biological replicates per group), the final cell suspension was adjusted to a concentration of 1000 cells/μL and loaded onto the 10x Genomics Chromium Controller, to generate single-cell gel beads in emulsion (GEMs) for downstream cDNA synthesis and library preparation using the 10X 3′ Single-Cell RNA-Seq V3.1 kits (10x Genomics, Pleasanton, CA, USA). Library concentrations were determined using the Qubit Fluorometric Quantitation method (Thermo Fisher Scientific, Waltham, MA, USA), and quality was assessed using the Agilent TapeStation system (Agilent, Santa Clara, CA, USA). Libraries were pooled and sequenced on the NovaSeq S4 2×100 platform (Illumina, San Diego, CA, USA) at ~20,000 reads per cell at the UCLA Broad Stem Cell Research Center. Raw sequencing reads were submitted to the Gene Expression Omnibus (GEO) with accession number GSE291555.

Sequencing reads were aligned to the mouse reference genome (mm10), and gene–cell count matrices were generated using Cell Ranger 7.1.0 (10x Genomics, Pleasanton, CA, USA). Downstream data analysis was performed using Seurat version 4.0.3^65^. Single cells were filtered according to thresholds of 200 to 5000 genes per cell, a maximum of 20,000 unique molecular identifiers (UMIs), less than 10% mitochondrial gene expression, and less than 60% ribosomal gene expression (Supplementary Fig. 12). Gene counts were normalized using Seurat’s default log normalization method via the NormalizeData function. Cells from all 4 single-cell RNA sequencing samples (2 replicates per EV or control treatment) were projected onto 2 dimensions by using Uniform Manifold Approximation and Projection (UMAP) based on the top 30 principal components of the gene expression profiles and clustered by using the Louvain clustering method (Supplementary Fig. 9). Cell type identification was carried out by evaluating the expression of known cell type markers within each identified cluster (Supplementary Fig. 8).

After cell type identification, differential gene expression analysis was performed using the FindMarkers function in Seurat, employing the non-parametric Wilcoxon rank-sum test to compare gene expression between EV-treated and control cells. Differentially expressed genes (DEGs) were identified by comparing each EV-treated sample (2 biological replicates) to both control samples. Overlapping genes between these comparisons that met an adjusted *P*-value threshold (Bonferroni correction) of <0.05 were selected. Among the selected genes, those common between the 2 biological replicates that exhibited consistent fold change direction were considered significant DEGs. For visualization, the average log fold change (logFC) between biological replicates was calculated, and adjusted *P* values from both replicates were combined using Stouffer’s method from the Metap package in R. Next, to understand the biological importance of the identified DEGs pathway enrichment analysis was done utilizing Hallmark database in EnrichR^59^. Pathways were considered significant at FDR<0.05.

### Hydrogel

Pluronic F-127 (Sigma Aldrich) and hyaluronic acid (Pharma Grade 80 from Novamatrix, with an average molecular weight of 620 kDa to 1.15 MDa) were used to prepare the hydrogel. A 50% (by wt) solution of Pluronic F-127 was prepared by dissolving 5 grams of the polymer in 10 mL of ice-cold Milli-Q water, followed by overnight incubation at 4 °C under continuous agitation to ensure complete dissolution. Separately, a 4% (by wt) solution of hyaluronic acid was prepared by dissolving 4 mg of the polymer in 10 mL of Milli-Q water at room temperature, also left overnight under continuous agitation. Both solutions were sterilized by filtration, and the final hydrogel compositions were obtained by mixing the 2 solutions in appropriate proportions. The prepared hydrogels were stored at 4 °C until use.

Hydrogel samples were prepared and immediately loaded between the parallel plates of a temperature-controlled rheometer (Anton Paar MCR302). A small-amplitude oscillatory shear test was conducted within the linear viscoelastic region by applying a sinusoidal strain across multiple frequencies. The storage modulus (G’) was recorded at each frequency, and values within the linear range were averaged to determine the elastic modulus. All measurements were performed in triplicate.

To assess hydrogel stiffness, an Instron 5542 mechanical tester was used. Samples were compressed at a rate of 1 mm min^−1^, and Young’s modulus was calculated from the slope of the linear region corresponding to 0–10% strain.

#### *In vitro* cumulative release of EVs from the hydrogel

For cumulative EV release studies, Flamma 675–labeled EV-loaded hydrogels were incubated in PBS at 37°C under gentle shaking. At predetermined time points, aliquots of PBS were collected and immediately replaced with fresh PBS to maintain a constant volume. Fluorescence intensity was measured using a plate reader, and EV concentration was quantified using a calibration curve generated from known concentrations of labeled EVs. The diffusion coefficient of EVs from the hydrogel was determined by fitting the cumulative release data to an analytical solution of Fick’s second law of diffusion for a cylindrical geometry. Nonlinear regression analysis was performed using curve-fitting software, and the diffusion coefficient was extracted from the best-fit parameters. Model fit quality was assessed using the R^2^ value.

#### *In vivo* tracking of EV release from the hydrogel

For *in vivo* EV tracking, Flamma 675–labeled EVs were incorporated into the hydrogel matrix. Mice were anesthetized, and 100 μL of the hydrogel was injected into the neck region. Baseline fluorescence images were acquired immediately post-injection using a Lumina II *In vivo* Imaging System (IVIS, PerkinElmer, Hopkinton, MA). Subsequent IVIS imaging was performed at designated intervals up to 48 hours under consistent imaging settings. Fluorescence signal intensity within the region of interest was quantified using Living Image software (Revvity, Waltham, MA) to assess EV retention and *in vivo* release kinetics.

### miR-302 transfection

Human CD4^+^ T cells were isolated as previously described. The miR-302 mirVana^®^ miRNA mimic (4464066, Thermo Fisher Scientific) was combined with Lipofectamine RNAiMAX in a 1:1 ratio to form complexes according to the manufacturer’s instructions and incubated for 5 minutes at room temperature before being added to the activated T-cell cultures. The mirVana^™^ miRNA Mimic Negative Control #1 (4464058) was used as a negative control. Changes in CDK8/19 expression were assessed after 3 days by PCR.

### Real-Time PCR

Total RNA was extracted from CD4^+^ T cells using TRIzol^™^ reagent (Thermo Fisher Scientific) and purified with the PureLink^™^ RNA Mini Kit, following the manufacturer’s protocols. RNA concentration and purity were assessed using the 260/280 nm absorbance ratio. Complementary DNA (cDNA) was synthesized from 1 μg of RNA using the Maxima First Strand cDNA Synthesis Kit for RT-qPCR (Thermo Scientific), with reactions performed at 25 °C for 10 minutes, 50 °C for 15 minutes, and terminated at 85 °C for 5 minutes. Quantitative real-time PCR (qRT-PCR) was conducted on the QuantStudio^™^ 5 system using the PowerUp^™^ SYBR^™^ Green Master Mix for qPCR (Applied Biosystems) in 10-μL reactions, containing 5 μL of master mix, 1 μL of primer pair (10 μM), 1 μL of cDNA (40 ng), and nuclease-free water. Primer sets specific for β-actin (HP204660), CDK8 (HP205183), and CDK19 (HP211372) were purchased from OriGene Technologies. Thermal cycling included 50 °C for 2 minutes, 95 °C for 2.5 minutes, followed by 40 cycles of 95 °C for 15 seconds and 60 °C for 1 minute, with fluorescence data collected during the annealing/extension step. Relative gene expression was calculated using the ΔΔCt method, normalized to ACTB.

### Adoptive-transfer T1D *in vivo* experiments

To evaluate the therapeutic effect of the designed EV hydrogel system in T1D, an adoptive transfer model was used. BDC2.5 CD4^+^ T cells were isolated from 6- to 8-week-old BDC2.5 NOD mice (004460, Jackson Lab) and activated for 3 days on plates coated with anti-CD3 (8 μg/mL) and supplemented with anti-CD28 (2 μg/mL) and IL-2 (50 U/mL). Subsequently, 200,000 activated T cells were adoptively transferred intravenously (i.v.) into NOD-scid mice (001303, Jackson Lab) via retro-orbital injection. On the day of T-cell transfer, control mice received no treatment, while experimental groups were subcutaneously injected with either 100 μg of iPSC EVs in PBS, 100 μL of hydrogel alone, or 100 μL of hydrogel encapsulating 100 μg of iPSC EVs. Injections were administered subcutaneously at the dorsal neck region. Blood glucose levels were monitored daily, and mice were considered diabetic when their blood glucose exceeded 250 mg/dL for 2 consecutive days.

For Treg analysis, 5 mice per group were sacrificed 10 days post-adoptive transfer. Draining, non-draining, and pancreatic lymph nodes, as well as spleens, were collected, dissociated into single-cell suspensions, and analyzed by flow cytometry. Briefly, cells were incubated in ACK lysis buffer (Gibco) for 5 minutes at room temperature to lyse red blood cells. Non-specific binding was blocked using TruStain FcX^™^ anti-mouse CD16/32 antibody (101320). Viability was assessed using the Zombie NIR Fixable Viability Kit (423105). Surface staining was performed using PE/Cyanine7 anti-mouse CD45 (103114), Alexa Fluor 488 anti-mouse CD3 (100210), Brilliant Violet 605 anti-mouse CD4 (100548), and Brilliant Violet 785 anti-mouse CD25 (102051) antibodies. For intracellular staining, cells were fixed and permeabilized using the FOXP3 Fix/Perm Buffer Set and stained with PE anti-mouse FOXP3 antibody (126404) alongside the Mouse IgG1, κ Isotype Control (Clone MOPC-21, 400130). All antibodies were purchased from BioLegend. Data acquisition was performed using a ThermoFisher Attune NxT Flow Cytometer, and analysis was carried out using FlowJo v10 software.

### Pancreatic tissue processing and beta-cell mass quantification

Pancreatic tissue samples were collected from the tail of the pancreas and fixed in 4% formaldehyde for 24 hours as previously described^66^. A spleen sample was also collected for comparison. Following fixation, the tissues were embedded in paraffin and sectioned at a thickness of 4 μm for subsequent analysis. Hematoxylin and Eosin (H&E) staining was performed according to an established protocol^67^. For insulin staining, rehydrated tissue sections were first blocked with 3% H_2_O_2_ in 10% methanol for 30 minutes at room temperature, followed by blocking with Tris B buffer for 1 hour. The sections were incubated overnight at 4 °C with guinea pig anti-insulin antibody (1:100; Abcam) diluted in Tris B. The next day, sections were incubated with biotinylated donkey anti-guinea pig secondary antibody (1:1000; Jackson ImmunoResearch) for 1 hour at room temperature. ABC Peroxidase solution (VECTASTATIN ABC Peroxidase PK-4000, Vector Laboratories) was applied for 1 hour, and staining was developed using DAB substrate solution for 30 to 50 seconds under microscopic observation. After rinsing in water, sections were counterstained with hematoxylin (Thermo Fisher Scientific), dehydrated through graded ethanol and toluene, and mounted using Permount mounting medium (Thermo Fisher Scientific).

For beta-cell mass quantification, tissue sections were scanned at 20x magnification using an APERIO scanner (Leica Biosystems). Beta-cell mass was quantified using the HALO Image Analysis Platform (Indica Labs). The beta-cell area, defined as the insulin-positive area, was expressed as a percentage of the total pancreas area. Total beta-cell mass was calculated by multiplying the insulin-positive area by the mouse’s pancreas weight.

### Statistical analysis

One-way ANOVA with Tukey’s post-hoc test or the Kruskal-Wallis rank sum test was used to assess significant differences across multiple samples. Student’s *t* tests were applied for comparisons involving 2 groups.

## Data Availability

All data supporting the findings of this study are available within the main text and Supplementary Information. The numerical source data underlying all figures and graphs in the main text (Figs. 1–6) and Supplementary Figures are available via the Figshare repository and can be accessed at [insert Figshare DOI upon publication]. Single-cell RNA sequencing data have been deposited in the NCBI Gene Expression Omnibus (GEO) under accession number GSE291555. All additional datasets generated and analyzed during the current study are available from the corresponding author upon reasonable request.
